# Teamwork: Improved eQTL Mapping Using Combinations of Machine Learning Methods

**DOI:** 10.1371/journal.pone.0040916

**Published:** 2012-07-24

**Authors:** Marit Ackermann, Mathieu Clément-Ziza, Jacob J. Michaelson, Andreas Beyer

**Affiliations:** 1 Biotechnology Center, Technical University Dresden, Dresden, Germany; 2 Center for Regenerative Therapy Dresden, Dresden, Germany; Mount Sinai School of Medicine, United States of America

## Abstract

Expression quantitative trait loci (eQTL) mapping is a widely used technique to uncover regulatory relationships between genes. A range of methodologies have been developed to map links between expression traits and genotypes. The DREAM (Dialogue on Reverse Engineering Assessments and Methods) initiative is a community project to objectively assess the relative performance of different computational approaches for solving specific systems biology problems. The goal of one of the DREAM5 challenges was to reverse-engineer genetic interaction networks from synthetic genetic variation and gene expression data, which simulates the problem of eQTL mapping. In this framework, we proposed an approach whose originality resides in the use of a combination of existing machine learning algorithms (committee). Although it was not the best performer, this method was by far the most precise on average. After the competition, we continued in this direction by evaluating other committees using the DREAM5 data and developed a method that relies on Random Forests and LASSO. It achieved a much higher average precision than the DREAM best performer at the cost of slightly lower average sensitivity.

## Introduction

The analysis of ‘genetical genomics’ data is an important step towards a systems-level understanding of molecular genetics data. It seeks to describe how natural genetic variability influences gene expression on a genome-wide level. Loci that are linked to the expression variation of a gene are called expression quantitative trait loci (eQTL). The advantage of this kind of analysis is its ability to elucidate causal regulatory relationships between genes without the need to actively perturb the system using e.g. gene knock-outs or knock-downs [1]–[4]. It is therefore applicable in situations where *in vivo* perturbations are not easily achieved. More importantly, it provides a much more realistic scenario than engineering non-physiological over- or under-expression of individual genes using genetic manipulations.

Much effort has been invested in the development of approaches for (e)QTL analysis [5]–[7]. Methods for eQTL mapping can be divided into univariate and multivariate (or single-marker versus multi-marker) mapping methods. The former infer the relationship between each single marker and a gene’s expression separately ignoring the effect of other markers, whereas the latter investigate the joint effects of genetic variations on the expression trait [5], [8], [9]. Multi-marker methods regard eQTL mapping as a feature selection problem: the expression of genes is predicted (explained) using a set of genetic markers [9]. Each marker is scored with respect to how informative it is for the prediction task. These methods often rely on penalized regression algorithms such as LARS [10] or machine learning techniques, e.g. Random Forests [11], which is based on an ensemble of regression trees. We have previously shown through investigation of both simulated and experimental data that multi-marker mapping methods clearly outperform single-marker methods [8].

The field of ensemble learning comprises all approaches in which a collection of possibly weak prediction models, so-called base learners, are combined to a robust and powerful model. The concept rests on the observation that combining disparate prediction algorithms has the potential to markedly improve prediction results [12]. Variants of ensemble methods include model averaging techniques and committee methods. For example, in Bayesian model averaging each prediction model contributes to the final model with a weight being proportional to its posterior distribution. On the other hand, bagging and Random Forests, both of which fall in the category of committee methods, obtain their final prediction as the majority vote (in classification) or average prediction (in regression applications) of all single learners. Boosting is considered as committee method as well, although the base learners evolve over time and each cast a weighted vote [13].

There is a need for systematically comparing the performance of eQTL mapping methods under different scenarios to reveal which approach works best in which context. However, due to the lack of trusted gold-standard gene-regulatory networks, it is not straightforward to evaluate the methods using real data [8]. The “DREAM5 SYSGEN A – In-silico network challenge” was set up to provide synthetic data that mimic the structure of real gene-regulatory networks, facilitating the assessment of the accuracy and sensitivity of eQTL mapping approaches that are currently used by the community.

We have decided to address this challenge using ensemble approaches. In particular, we developed filtered and unfiltered committees by combining the predictions of several machine learning methods. As part of the DREAM5 PLoS One collection, we present an overview of the results obtained with several machine learning approaches and show that any combination of the methods outperforms the individual methods. We also show that our proposed approaches lead to a much higher average precision than the other DREAM challenge contributions, at the cost of slightly lower average sensitivity. Finally, we discuss the importance of precision compared to sensitivity in eQTL mapping.

## Methods

Multivariate mapping approaches such as Random Forests [11], the LASSO [14] or the Elastic Net [15] either choose a small subset of predictors or shrink some of their coefficients so that they drop out of the model. These approaches are based on different criteria, implying different underlying biological assumptions. For example, the Elastic Net includes groups of correlated variables into a linear model, while the LASSO will only include one predictor out of this group, namely the one which shows the highest correlation with the response. Additionally, both the Elastic Net and the LASSO assume that the measured trait is a linear combination of the genotypes, and do not implicitly allow for epistatic interactions. In contrast, tree-based methods such as Random Forests allow for epistatic interactions between the genotypes selected in the model. This distinction is not as relevant to the data in this paper, since it was simulated without the inclusion of these kinds of genetic interactions. However, when applying the method to real data, such considerations are important in order to accurately capture the underlying biological processes.

Since the true model is unknown, and will be different for different genes, we decided to combine several multivariate eQTL mapping methods into committees in order to capture different regulatory mechanisms and average out false positive findings due to noise in the data. We tested different committees of the following methods: Random Forests with two different variable importance measures: permutation importance and selection frequency; the LASSO and the Elastic Net.

### Random Forests Selection Frequency (RF.sf) and Random Forests Permutation Importance (RF.pi)

Random Forests [11] are ensembles of decision trees. Each tree in the forest is fit to a bootstrap sample of the complete dataset, and each split in the trees is performed by selecting the best split from a randomly selected subset of predictor variables. In the case of eQTL mapping, these predictors are genotype markers. In this work, we used two importance measures that are indicative of eQTL. The first, selection frequency (RF.sf), is simply the frequency at which a marker was selected as the best splitting variable throughout the forest. The second is the permutation importance (RF.pi), which reflects the average decrease in predictive accuracy observed when a marker is permuted randomly.

We used the reference implementation of Random Forests in R [16] for the Random Forests mapping [17]. We grew forests with 5,000 trees, the *mtry* parameter (number of variables randomly sampled as candidates at each split) was set to the default (one third of the total number of predictors) and the minimum node size was also set to the default of 5. We then extracted unscaled permutation importance measures (RF.pi) and selection frequencies (RF.sf) from the forests for use as the scores for each predictor.

### LASSO Coefficient

Tibshirani developed the least absolute shrinkage and selection operator (LASSO) to improve variable selection for linear regression with regard to prediction accuracy and interpretation [14]. The standard approach for estimating the coefficients of a linear regression is ordinary least squares (OLS). However, when the number of predictors exceeds the number of observations, as is the case in eQTL mapping, OLS fails to select the subsets of predictors that are the most predictive. LASSO overcomes this problem by imposing a constraint on the coefficients which is based on the L_1_ norm. LASSO thus tends to set many regression coefficients to 0 in order to retain the most important predictors and to produce an accurate and interpretable model [14].

We used the LASSO implementation from the elasticnet package [15] for R [16] by setting the quadratic penalty λ to 0.001. For each gene, we took the absolute value of the LASSO coefficients for a fit performed with *s* (fraction of the L_1_ norm) determined by 10-fold cross-validation, with an imposed minimum of 0.25. These coefficients were used as the importance score for each predictor.

If there is a group of correlated predictors that all predict the expression trait equally well, LASSO will give a high importance score to only one of them (the predictor most highly correlated with the response). All other predictors in the group will drop out of the model.

### Elastic Net coefficient (ElNet)

Elastic Net is a combination of LASSO and Ridge regression, which uses an L_2_ regression penalty. It has been shown that compared to the LASSO, the Elastic Net is more suited for situations in which the number of predictors greatly exceeds the number of observations [15]. In contrast to LASSO, Elastic Net will always select an entire group of predictors explaining the trait equally well and thus would give large regression coefficients to a whole set of correlated genotype markers. This results in substantial differences with respect to how these two methods deal with markers in linkage disequilibrium (LD): markers that are physically close on the genome will always be correlated. Whereas LASSO will only give one predictor in such a group a nonzero coefficient, Elastic Net will distribute nonzero coefficients among predictors in the group.

Again, we used the absolute coefficients of the best model (found by ten-fold cross validation with the elasticnet package) as importance scores for the predictors, this time setting λ to 1.

### Unfiltered Committees of Predictions

Each method assigns some kind of importance score to each predictor – gene pair. We combined these in committees by averaging the scaled and centered scores. We tested different combinations of methods leading to slightly different performance on the DREAM data. Of particular interest is using all importance scores:
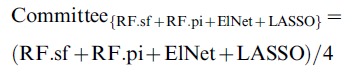



and using only RF.sf and LASSO:
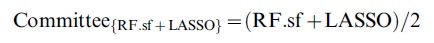



### Filtered Committee

We also investigated a modified version of our committee approach, which seeks for a very sparse solution, i.e. only a very limited number of regulators per gene. In this case, the scores from all methods except the LASSO were scaled and an average score was calculated for each regulator – target gene pair. Subsequently, each average score was set to zero if the corresponding LASSO score was equal to zero. In other words, only variables that were chosen by the very sparse LASSO algorithm got a nonzero final score.
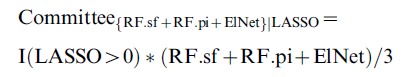



This filtering leads to a very different treatment of markers in linkage disequilibrium. Whereas the unfiltered scoring above will give all markers in a linked region relatively high scores, the filtering results in the selection of only one marker from this region.

### Evaluation of the Performance

In the dream challenge, the area under the Receiver Operator Characteristic (ROC) curve (AUROC) and the area under the precision-recall curve (AUPR) were used to evaluate the performance of the prediction methods [18], [19]. The ROC curve shows how the proportion of correctly classified positive instances (True Positive Rate, TPR, also named sensitivity or recall) varies with the proportion of incorrectly classified negative instances (False Positive Rate, FPR) [20], [21]. The AUROC therefore represents i) the average specificity across all sensitivities, ii) the average sensitivity across all specificities, and iii) the probability of an existing edge to have a higher score than a non-existing one [22], [23]. The precision-recall curve compares the fraction of retrieved positive instances (correctly predicted interactions among all predictions) to the fraction of true positives (correctly predicted interactions among all interactions). The AUPR therefore reflects the average precision of predictions across all recall thresholds [24].

## Results

### The Systems Genetics Challenge Problem

The DREAM5 systems genetics *in silico* network challenge A consisted of reconstructing a gene-regulatory network from synthetic genetic variation and gene expression data. The data were generated after the following scheme (see http://wiki.c2b2.columbia.edu/dream/index.php/D5c3 and [25] for details): fifteen different directed gene-regulatory networks consisting of 1,000 genes each were simulated following a scale-free out-degree and an exponential in-degree distribution. The networks varied both in the number of samples (100, 300 and 999; corresponding to the 3 different sub-challenges A100, A300 and A999, respectively) as well as the total number of edges. Network 1 in each sub-challenge comprised about 2,000 edges; the number of edges increased to ∼5,000 for network 5 in each sub-challenge.

The simulated genotypes of 1,000 markers, each corresponding to a mutation in exactly one of the 1,000 genes, imitate the architecture of recombinant inbred lines (RIL). RILs are lines derived from a cross between two genetically distinct inbred parental lines, and are homozygous at every locus as a result of inbreeding for multiple generations. Each of these RILs is homozygous for the allele of one of the parents (i.e. each RIL genotype vector can be coded in a 0/1 scheme), and each RIL has inherited different combinations of parental alleles. The RILs constitute a genetically randomized population, meaning that the gene expression pattern of each RIL is the result of a different multifactorial genetic perturbation (quoted from the DREAM web-site: http://wiki.c2b2.columbia.edu/dream/index.php/D5c3) [26]. Each mutation occurred either in the promoter or the coding region of the corresponding gene, with probabilities of 0.25 and 0.75, respectively, which corresponds to mimicking *cis*- and *trans*-effects. The former will affect the expression level of the gene itself, while the latter modify the effect of the gene on the transcription levels of its down-stream targets. The genotypes of the 1,000 markers were evenly distributed on 20 chromosomes and local linkage between adjacent positions on the chromosomes was taken into account during the simulation process. Finally, gene expression levels of each gene were simulated at a steady state of a dynamical model built from a set of ordinary differential equations (ODEs). The parameters of these ODEs define the level of the activation and repression effects of the genes on their targets as well as the influence of genetic variants and the noise levels. Details of the ODE setup can be found in [26].

As in an eQTL study, the aim of the DREAM5 SYSGEN A challenge was to retrieve the regulatory relationships of each network using i) the simulated gene expression levels of the 1,000 genes in each RIL, and ii) the simulated genotype data of the RILs. Results had to be presented as an ordered list of edges between pairs of genes, where the edge scores were only used for ranking and did not necessarily represent any kind of statistical significance of the inferred edges.

### Assessment of Individual Mapping Methods

**Figure 1:**
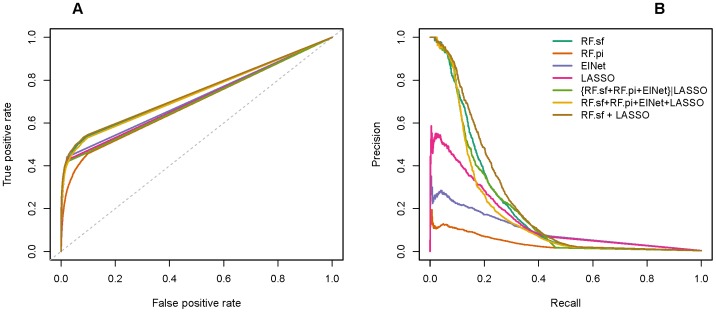
ROC and precision-recall curves for individual multivariate methods and combinations thereof. ROC curves and precision-recall curves obtained for the prediction of one representative network with an intermediate number of edges and 300 RILs (network 3 of sub-challenge A300). We compare the performance of all four individual methods (RF.sf, RF.pi, LASSO and ElNet), the combination of Random Forests selection frequency and LASSO (RF.sf+LASSO), the combination of all four approaches (RF.sf+RF.pi+ElNet+LASSO) as well as the filtered committee we submitted to the challenge ({RF.sf+RF.pi+ElNet}|LASSO). Left: ROC curves. Right: precision-recall curves. The differences in performance between the methods are more apparent in the precision-recall curves.

Following the conclusion of the DREAM5 challenge, the reference networks were released. We used these data to evaluate the performance of the four multivariate eQTL mapping methods comprising our committee approach, individually and in combination. Figure 1 shows the ROC and precision-recall curves for all four individual methods (RF.sf, RF.pi, LASSO and ElNet), the combination of all methods (RF.sf+RF.pi+ElNet+LASSO), the filtered committee ({RF.sf+RF.pi+ElNet}|LASSO) and the best performing of all combinations (RF.sf+LASSO). Notably, all individual methods are outperformed by at least one combination of approaches both in terms of ROC and precision-recall curve (Figure 1). However, there are striking differences between the individual approaches. RF.sf outperforms the other methods by far regarding the precision-recall curves. Its performance is very close to most of the combinations of approaches. Still, combining RF.sf with LASSO or other methods can improve the precision at a given level of recall (Figure 1B). RF.pi, ElNet and LASSO do not reach precisions above 0.6 even when considering only the first few top ranking edges. This suggests that these methods lead to a relatively large fraction of false positive findings among the top-scoring edges. Moreover, both LASSO and ElNet are methods that aim to limit the number of features used in the model. This restrictiveness in terms of the number of predicted edges is reflected by the kinks in the ROC curves obtained when using these methods (Figure 1A).

**Figure 2:**
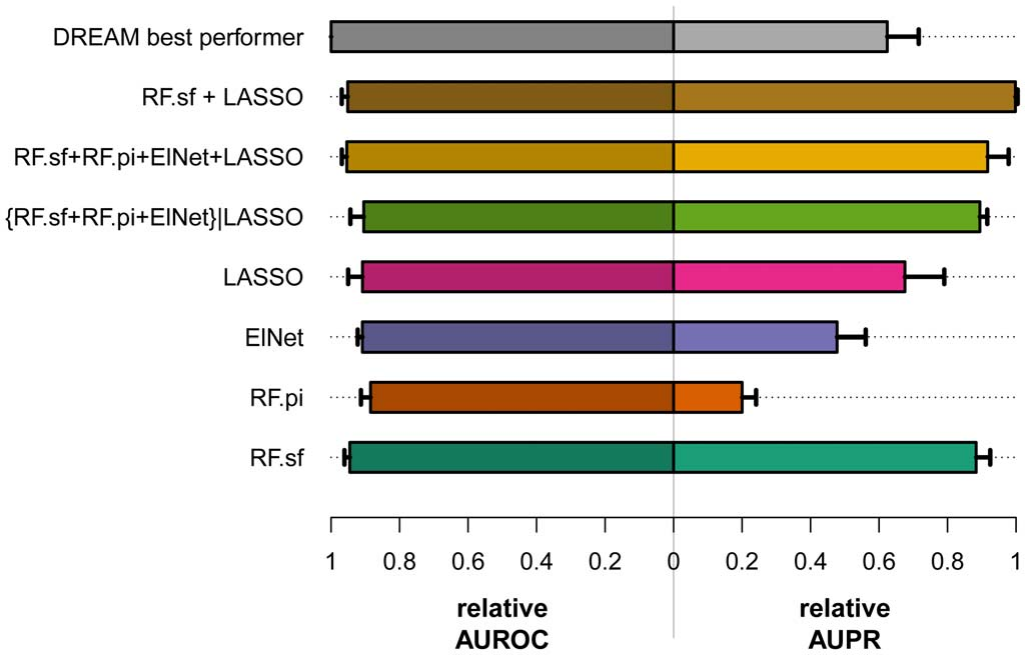
AUROC and AUPR for all tested methods. For each of the 15 networks of the DREAM5 SYSGEN A challenge, we evaluated the performance of the different methods using the AUROC and AUPR as metrics. To better compare AUROC and AUPR values, they were scaled to the maximum value obtained across methods for each network. Results were then summarized over all 15 networks. The bars show the mean AUROC (left-oriented bars) and AUPR (right-oriented bars) per method, error bars indicate one standard deviation. RF.sf+LASSO outperforms the DREAM best performer and all our tested approaches in terms of AUPR. Differences between the methods on AUROC values are less pronounced.

**Figure 3:**
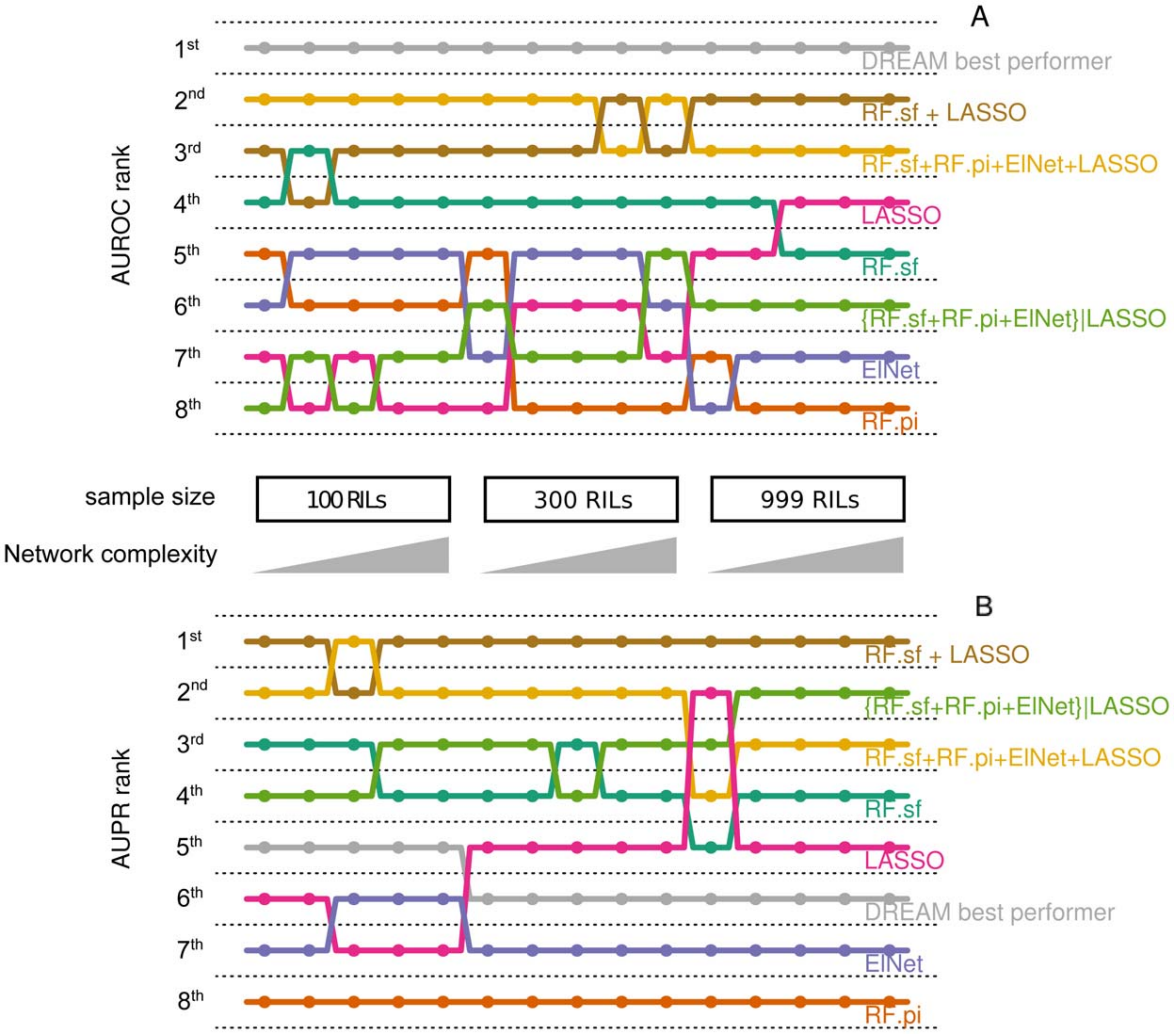
Rankings of all methods based on AUROC and AUPR. For each of the 15 networks of the DREAM5 SYSGEN A challenge (5 for each sample size), the performance of the different methods was ranked using both the AUROC (A) and the AUPR (B). For each method, ranks are plotted horizontally across all networks. Sample sizes (number of RILs) and network complexity (number of edges) used for simulating the network are shown between the panels. While the DREAM best performer always ranks best based on the AUROC (Panel A), RF.sf+LASSO ranked first in all but one network based on AUPR (Panel B).

The organizers of the DREAM challenge used both the area under the precision recall curve (AUPR) and the area under the receiver operating characteristic curve (AUROC) to assess how well the predicted networks approximate the gold standard networks [18], [19]. RF.sf almost always achieved better rankings in both AUPR and AUROC than the other three individual methods (Figures 2 and 3). Interestingly, the superiority of the performance of the RF.sf compared to the other individual methods increases with decreasing number of RILs (Figure S1); in other words, the RF.sf outperforms other mapping methods in particular when the number of samples is relatively small, a scenario that closely reflects the real-world constraints of eQTL studies [8]. RF.sf also achieved a better average precision (based on AUPR) than the best DREAM participant.

### Prediction Using the Filtered Committee: {RF.sf+RF.pi+ElNet}|LASSO

The method we proposed for the challenge was a filtered committee of the four tested multivariate eQTL mapping methods ({RF.sf+RF.pi+ElNet}|LASSO). This approach was designed to identify a small number of regulators per gene with high accuracy. It consists in a combination of 3 variable importance measures (two from Random Forests and one from the Elastic Net) filtered based on the presence of a nonzero LASSO coefficient. The AUPR obtained by our filtered committee approach was the highest among all the methods competing in the challenge, and for all networks (Figures 3B and S1). Moreover, the AUPR across the 15 networks was on average 46.1% (sd 20.8%) higher than the DREAM best performer, whereas the AUROC was on average 9.53% (sd 3.9%) lower. This shows that, compared to the best performer of the DREAM challenge, on average the relative gain in precision of the {RF.sf+RF.pi+ElNnet}|LASSO method at any given recall rate is much greater than the average relative loss of sensitivity. The main weakness of this approach was its drop in the slope of the ROC curves (Figure 1A). One of the reasons the filtered committee method performed worse than the other methods may have been the sparseness of its predictions. The {RF.sf+RF.pi+ElNet}|LASSO method predicted indeed at most 23,361 edges in the studied networks (Figure S2). However, the top 100,000 predictions were used to evaluate the performance, and therefore, random edges were added to the predictions produced by the {RF.sf+RF.pi+ElNnet}|LASSO method to reach 100,000. To reduce sparseness of the prediction, we tested an unfiltered integration of the four different approaches, which led to better results in terms of AUROC and AUPR (Figures 2, 3 and S1).

### Combination of Two Methods Outperforms All Other Alternatives

We have investigated the predictions of the committees consisting of all the possible unfiltered combinations of the four methods. Most of our committees outperform the best DREAM performer in terms of AUPR at the cost of a slightly worse AUROC (Figures 2, 3 and S1, only the best unfiltered combinations are shown). Overall, we also observed that the committees outperform their constituent methods. The committee composed of the Random Forests selection frequency and the LASSO (RF.sf+LASSO) performed better than all other evaluated methods (Figures 1, 2, 3, Figure S1). Interestingly, RF.sf and LASSO were also the two individual methods that performed best among the four that we have evaluated. RF.sf+LASSO outperforms the DREAM best performer and all our tested approaches in terms of AUPR. The relative gain in AUPR increases with increasing sample size and network complexity (Figure S1). Compared to the best performer of the DREAM challenge, the combination of these two methods showed slightly lower average sensitivity (average AUROC decrease of 4.68%, sd 1.74%) but much higher average precision (average AUPR increase of 63.5%, sd 25.4%). This shows that the combination of a small number of machine learning methods has the potential to considerably improve prediction results, especially in terms of average precision.

## Discussion

In this article, we have tested several methods to reverse-engineer eQTL networks from synthetic expression and genotype data [5]–[7]. The merit of our approach resides in combining existing machine learning algorithms in committees. Since the predictions of the other challenge participants are not public, we cannot directly compare the precision-recall curves of our approaches to their results. However, the filtered committee we submitted to the DREAM5 competition achieved higher AUPR than any other competing method in the challenge. After the release of the DREAM5 gold standard networks, we continued working in this direction by testing other committees using the DREAM5 framework and identified methods that achieve much higher AUPR than the DREAM best performer at the cost of only slightly lower AUROC values.

**Figure 4:**
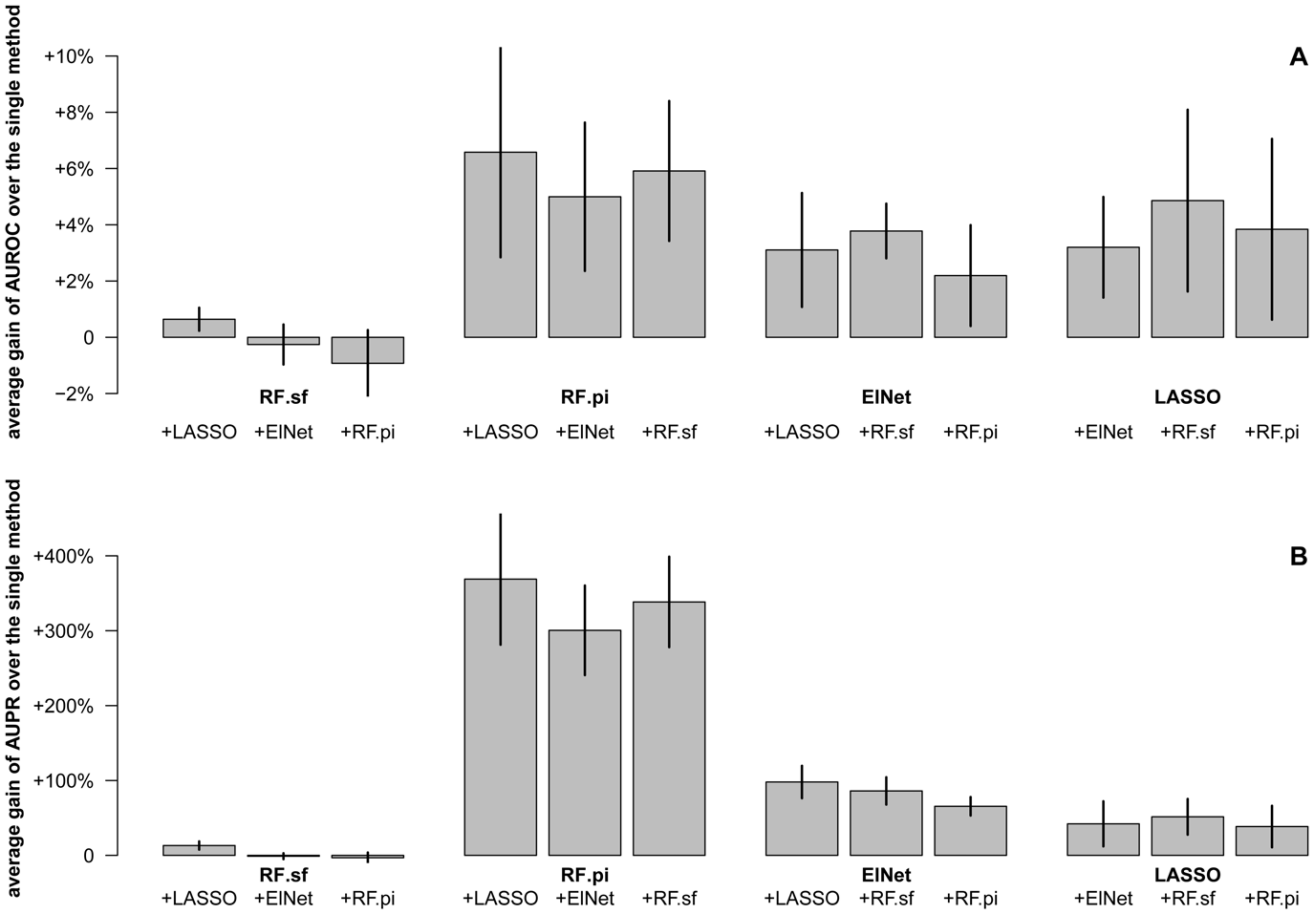
Gain in AUROC and AUPR for committees made of method pairs. We evaluated the committees composed of all possible pairs of the four single variable selection methods (RF.sf, RF.pi, ElNet and LASSO). In order to assess if committees were beneficial, we compared their performance to the performance of their constituent methods. For each combination of method pairs, we calculated the ratio of the AUPR and AUROC of the constituent methods over the AUPR and AUROC of the committee. We used this ratio to compute the gain of AUROC (A) and AUPR (B) obtained by the committees over the constituent methods and averaged this over the 15 networks of the DREAM challenge. Error bars represent the standard deviation. This figure shows that the committees are almost always more predictive than the constituent methods.

When the amount of training data is limited (as is the case in eQTL mapping), many models can explain the data equally well. In machine learning this is well known as the “small *n*, large *p* problem”: the number of samples is small compared to the number of parameters and thus, the system is underdetermined [27]. A model using all available parameters is likely to overfit the data, leading to a large variance in the predictions sensitive to small variations of the training data. On the other hand, using too few parameters will lead to high bias. Ensemble methods are widely used in machine learning, because they enable the simultaneous reduction of variance and bias, even when the size of the training data is small [27], [28]. In fact, the Random Forests method is itself a committee. Random Forests learns an ensemble of decision trees by varying the learning data, yielding stable models (low variance) with a minimized bias [11]. Consistent with the known superior performance of ensemble methods, we have previously shown that RF outperforms other eQTL mapping methods [8]. Here, we combined RF and other modeling techniques into committees, a type of ensemble [13]. We observed that these committee methods almost always performed better than their constituent methods (Figure 4).

When groups developing algorithms are also the ones validating them, the benchmark data and the assessment metrics can be biased (knowingly or not) in favor of the proposed algorithm [29]. A key aspect of the DREAM challenges is that the ‘ground truth’ data is obscured from the participants [29], resulting in a more objective assessment than most computational methods papers can provide. This makes the DREAM challenges a valuable tool for the computational biology community. Our approach had already proven its value within the context of the DREAM challenge itself (before the evaluation data was released). Here, we extended the analysis of committee methods and tested additional combinations of the learners in order to better understand the factors that explain the performance of our approach. This analysis revealed that Random Forests alone – which in itself is a committee method – performed almost as well as the combined approach that we chose for the challenge. Combining just two out of the four methods that we included in our initial committee (RF.sf+LASSO) yielded top performance. The role of LASSO may be to ameliorate the problem of linkage disequilibrium, i.e. given a linked region, LASSO identifies the marker within the region that is most likely associated with the expression of the target gene. LASSO could therefore be used for ‘fine mapping’ the causal locus.

The evaluation of the performance of the methods competing in the DREAM5 challenge relies on the AUROC and AUPR. The Receiver Operator Characteristic (ROC) curve shows how the fraction of correctly classified positive instances (True Positive Rate, TPR) varies with the fraction of incorrectly classified negative instances (False Positive Rate, FPR) [20], [21]. It has been argued that ROC curves are not reliable when there is a large skew in the class distribution; under this condition they strongly over-estimate an algorithm’s performance [30], [31]. In the case of gene-regulatory network reconstruction or eQTL mapping, the number of negative instances greatly exceeds the number of positive instances; i.e. the number of true interactions is only a small portion of the potential interaction space. This implies that large differences in the number of false positives (i.e. the number of incorrectly predicted interactions) may only slightly affect the FPR and therefore lead to small changes in the AUROC. In contrast to this, precision, which drives the AUPR, compares the number of false positives (incorrectly predicted interactions) to the number of true positives (correctly predicted interactions) and is thus more sensitive against small changes of the number of false positives when the number of true negatives (non-interacting pairs of genes) is large. Precision-recall curves are therefore considered as an alternative to ROC curves when the class distribution is skewed [21].

We showed that our approaches yield a much higher AUPR at the cost of a slightly lower AUROC than the other competing methods of the DREAM challenge. We argue here that in the case of eQTL mapping, the AUPR may better assess the performance of the competing methods, in the way that it penalizes the detection of false positive edges among the top scoring edges more heavily than the AUROC score. Indeed, in practice the prediction of a regulatory relationship is only the first step of the analysis. The predicted relationships can be used as a basis to study a biological process, or be validated in a follow-up experiment, or (more commonly) be integrated with other data to make biological inferences. Depending on the down-stream analysis, erroneous prediction of an interaction may be much more expensive than missing an interaction.

Data simulations are a well-established means to test new approaches for data analysis and compare them to state of the art methods in the field. However, the more complex the data to be analyzed, the more difficult it is to mimic these data with simulations. While the DREAM5 SYSGEN A data were designed to simulate the complex regulatory relationships between genetic loci and gene expression, there are several considerations missing from the data-generating model. Epistatic interactions between loci (non-additive effects) greatly complicate the structure of eQTL networks [32], [33]. The model underlying the DREAM5 SYSGEN A data includes multiplicative effects of the regulators on gene expression. However, true epistatic effects may also include other types of interactions, for example an XOR relationship between two loci. Additionally, in practice, methods have to be able to cope with missing data (in the genotyping as well as in the phenotyping of the RILs). Further, the ratio of strains being tested versus the number of markers is often lower than in the DREAM5 challenge, thus creating additional statistical complications [34]–[37]. Finally, by equating eQTL and gene loci (i.e. there are no intergenic regions in the simulated data), the DREAM challenge avoids the problem of finding the true causal polymorphism and relating it to the genomic feature driving the eQTL. This is arguably the most difficult part of any eQTL study and is vital for any biomedically beneficial result of the analysis. We believe that while the DREAM5 challenge is a good first step in developing methods to discover gene-regulatory networks from systems genetics data, there are some clear steps that could be taken to make the simulated data more closely mirror the characteristics of real-world data. It would be of interest to assess the performance of the kinds of methods we have described here on future community shared benchmarks that better reflect the complexity of eQTL mapping and also to integrate real data into the evaluation procedure [8].

## Supporting Information

Figure S1 Area under the ROC (AUROC) curve and area under the precision-recall (AUPR) curve for each of the 15 networks of the DREAM 5 SYSGEN A challenge. The bars show the AUROC (left-oriented bars) and AUPR (right-oriented bars) for each method and each netwrok. Top panel, 100 RILs. Middle panel, 300 RILs. Bottom panel, 999 RILs. Complexity of the networks (number of edges) increases from left to right in each panel.
(PDF)

Figure S2 Number of interactions predicted by the filtered committee ({RF.sf+RF.pi+ElNet}|LASSO) for each of the 15 networks of the DREAM5 SYSGEN A challenge. The challenge was divided into three sub-challenges with varying sample sizes (100, 300 and 999 RILs, respectively), and each sub-challenge consisted of 5 different networks with growing numbers of edges. The number of predicted interactions positively correlates with sample size and network complexity. For the evaluation of the challenge, the top 100,000 scoring interactions were considered. The {RF.sf+RF.pi+ElNet}|LASSO method was very restrictive in the number of predicted network edges. Since the {RF.sf+RF.pi+ElNet}|LASSO did not predict that many interactions for any network, the evaluators added random interactions.
(PDF)
