# Supplementary figures and images for: Teamwork: Improved eQTL Mapping Using Combinations of Machine Learning Methods
Source: PLoS One. 2012 Jul 24;7(7):e40916. doi: 10.1371/journal.pone.0040916 (PMC3404069; doi:10.1371/journal.pone.0040916)

increasing network complexity

100 RILs

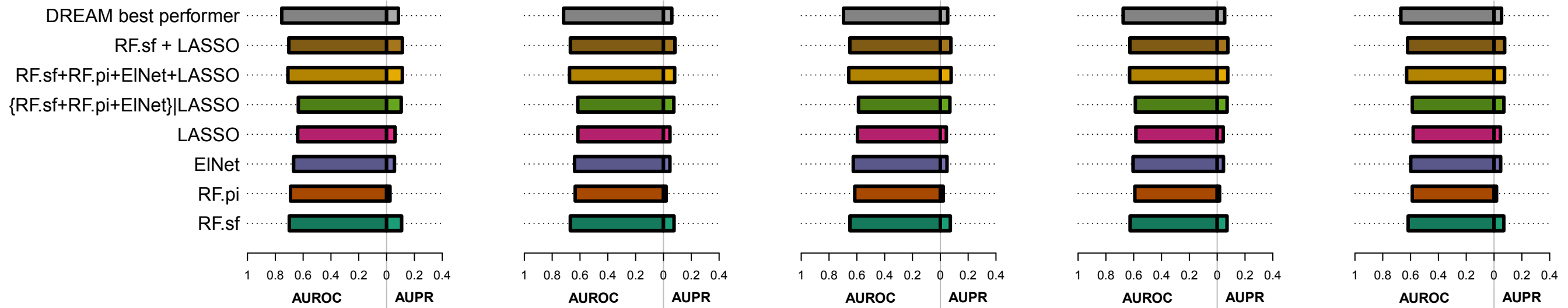

300 RILs

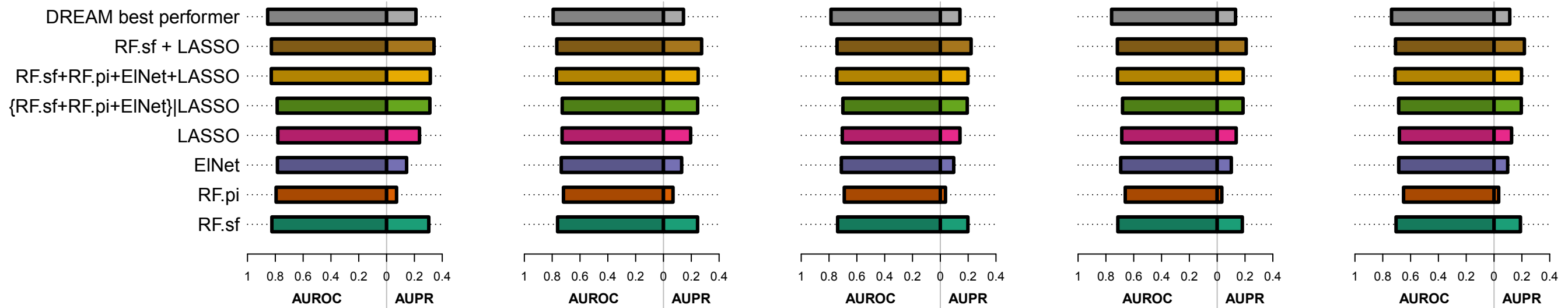

999 RILs

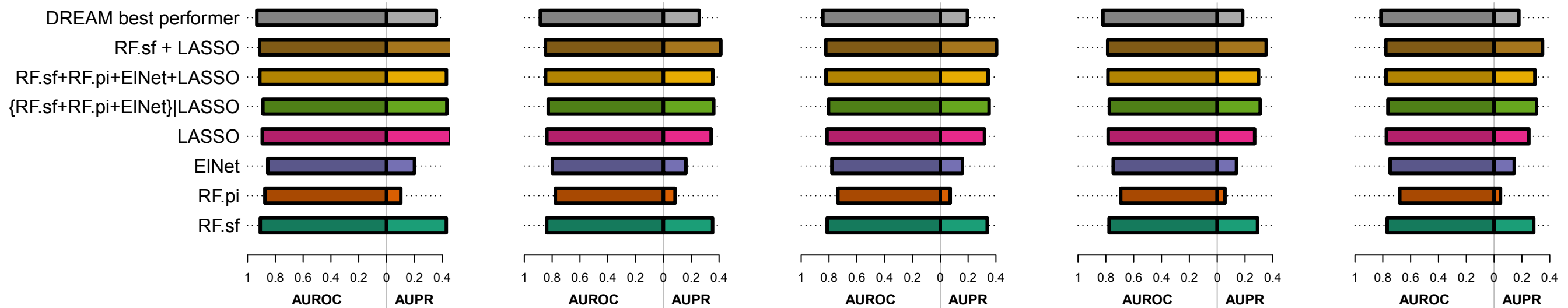

Supplement: Figure S1 — Area under the ROC (AUROC) curve and area under the precision-recall (AUPR) curve for each of the 15 networks of the DREAM 5 SYSGEN A challenge. The bars show the AUROC (left-oriented bars) and AUPR (right-oriented bars) for each method and each netwrok. Top panel, 100 RILs. Middle panel, 300 RILs. Bottom panel, 999 RILs. Complexity of the networks (number of edges) increases from left to right in each panel. (PDF) [file pone.0040916.s001.pdf]

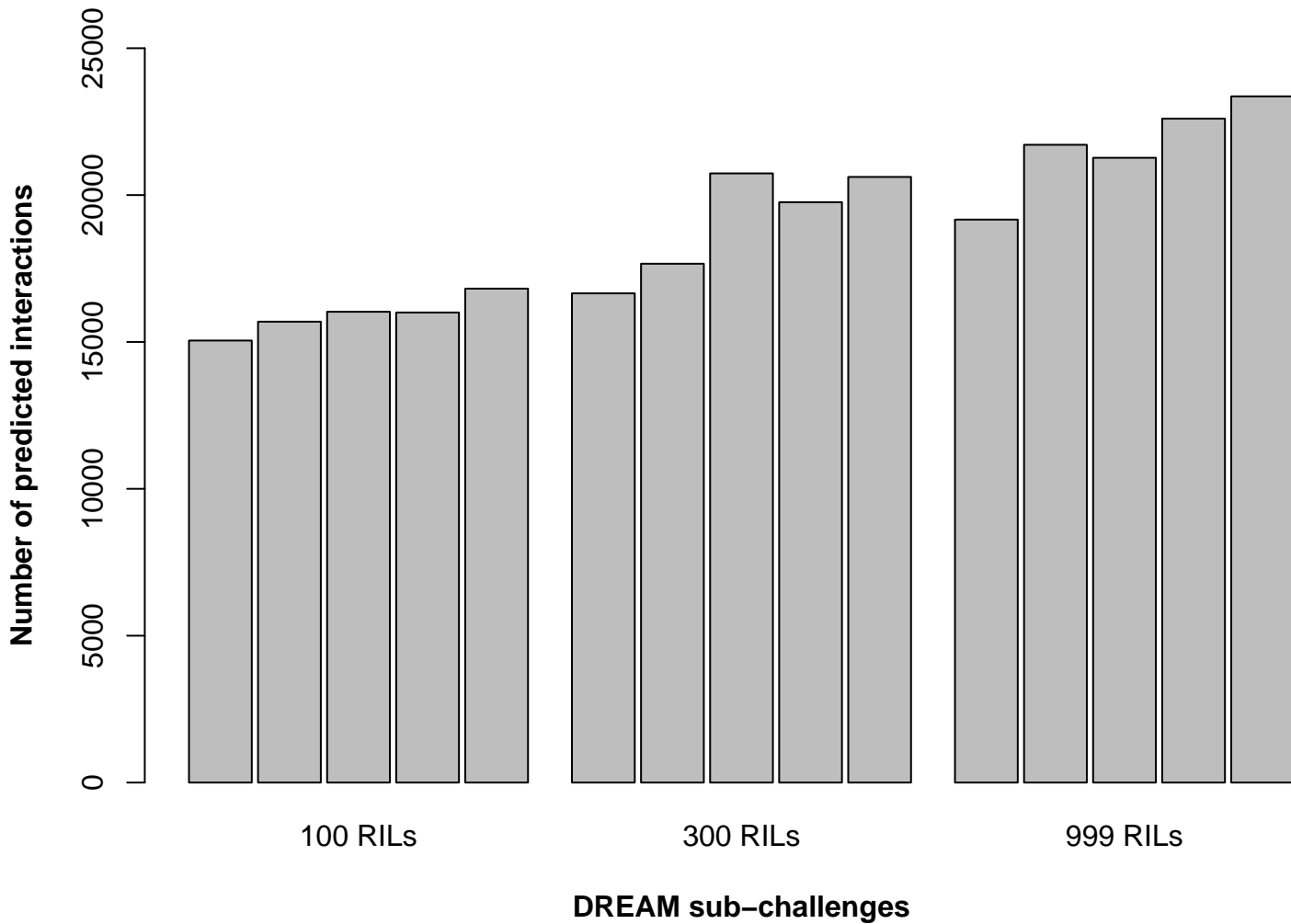

Supplement: Figure S2 — Number of interactions predicted by the filtered committee ({RF.sf+RF.pi+ElNet}|LASSO) for each of the 15 networks of the DREAM5 SYSGEN A challenge. The challenge was divided into three sub-challenges with varying sample sizes (100, 300 and 999 RILs, respectively), and each sub-challenge consisted of 5 different networks with growing numbers of edges. The number of predicted interactions positively correlates with sample size and network complexity. For the evaluation of the challenge, the top 100,000 scoring interactions were considered. The {RF.sf+RF.pi+ElNet}|LASSO method was very restrictive in the number of predicted network edges. Since the {RF.sf+RF.pi+ElNet}|LASSO did not predict that many interactions for any network, the evaluators added random interactions. (PDF) [file pone.0040916.s002.pdf]
